# Key factors supporting implementation of a training program for neonatal family- centered care – a qualitative study

**DOI:** 10.1186/s12913-019-4256-1

**Published:** 2019-06-19

**Authors:** Mirka Toivonen, Liisa Lehtonen, Sari Ahlqvist-Björkroth, Anna Axelin

**Affiliations:** 1 Department of Nursing Science, University of Turku, FI-20014 Turku, Finland; 2 Faculty of Medicine, University of Turku, Turku, Finland; 3 Hospital District of Southwest Finland, Department of Pediatrics Turku University Hospital, Kiinamyllynkatu 4-8, PL 52, 20521 Turku, Finland; 4 Department of Psychology and Speech-Language Pathology, Faculty of Social Sciences, University of Turku, FI-20014 Turku, Finland

**Keywords:** Family-centered care, NICU culture, NICU staff training, Reflective supervision, Neonatal intensive care units, Neonatal nursing

## Abstract

**Background:**

Traditionally, the care of infants in neonatal care units has been professionally centered, paying less attention to family support. In recent years, many interventions have been developed to improve family-centered care and thereby parent and infant outcomes. Understanding the key factors of implementation of these interventions would help improve clinical practice. The aim of this study was to describe the staff’s perceptions of the implementation of the Close Collaboration with Parents Training Program and to identify the barriers and facilitators of the implementation.

**Methods:**

A descriptive qualitative interview study was conducted in eight neonatal intensive care units in Finland. Nineteen unit managers and 32 nurses were interviewed after their unit had finished the 1.5-year training program. Data were analyzed using thematic content analysis.

**Results:**

Key factors facilitating the implementation of the training program were multidisciplinary commitment and the staff’s motivation to change their professional role to work as the parents’ facilitator. Observable benefits promoted the implementation, as well as experiential learning as a facilitation method. The role of mentor was remarkable as a facilitator. In addition, contextual elements such as support from leadership and proper timing were important.

**Conclusions:**

Implementation of family-centered care is facilitated by staff who is prepared to accept parents as partners and adopt a new professional role. Enough time for preparation, readiness for the change, solid support from the leadership, and a multidisciplinary approach are needed as well. Mentoring was found to be one of the key factors facilitating the change.

**Electronic supplementary material:**

The online version of this article (10.1186/s12913-019-4256-1) contains supplementary material, which is available to authorized users.

## Background

Family-centered care (FCC) is a philosophy emphasizing partnership between parents and health care staff [1]. The basic principles of FCC in neonatal intensive care units (NICUs) are unlimited parental presence and parental participation, shared responsibility and decision-making about the infant’s hospital care, and open communication between parents and staff [1, 2]. Increased parental presence and participation in infant care have led to better developmental outcomes of infants [3, 4], decreased parental anxiety and depression [5, 6], and resulted in more confident and informed parents [7]. Welch et al. (2015;2016) [8, 9] showed that a FCC intervention significantly improved both mother and infant long-term outcomes across neurobehavioral, psychological and physiological domains.

There are factors that have slowed down the implementation of FCC in everyday care practices. Traditional unit design and professionally-centered staff attitudes have been identified as barriers [1, 2]. Health care professionals may be uncertain about how to translate the principles of FCC into action [10, 11]. They may not know how to support parents in the collaborative partnership or what the expectations for the partnership are [11] because NICUs have traditionally been environments that are closed to parents. Many NICU nurses still rely on experience and tradition in their nursing practice [12], and changing their work identity from task-oriented to family-oriented can be challenging [10, 13, 14].

Several complex interventions have been shown to be effective in enhancing FCC in the NICUs. These include an educational–behavioral intervention program for parents [5], family-centered rounds [15], developmental care [16], family nurture intervention [8], and family-integrated care [17–20]. The evaluation of complex FCC interventions with randomized controlled trials design have been conducted in controlled environments that do not consider the impact of contextual factors in their implementation [21]. Therefore, implementation research is needed to understand why and how FCC interventions work and to identify factors affecting their implementation in clinical practice [21, 22].

FCC is a care philosophy based on comprehensive care culture. Therefore, any intervention has to be unit-wide and aim to change the attitudes of the NICU staff, not only single care practices [12]. Our intervention, the Close Collaboration with Parents™ Training Program, is targeted at the whole NICU staff, including doctors and nurses, and aims to facilitate the staff to work with the parents and promote FCC. The final goal of the training program is to change the care culture so that the parents are accepted as partners in infant care [23]. In this article, we report staff’s perceptions in eight hospitals on the implementation process and the key factors affecting it.

## Methods

### Aim

The purpose of this study was to explore the staff’s perceptions of the implementation of the Close Collaboration with Parents Training Program and to identify the key barriers and facilitators of the implementation.

### Study design

The study is a part of the evaluation of the effectiveness of the training program. The process evaluation [24] with focus group interviews was included in the study design to understand which factors affect the implementation of the training program.

### Setting

This study included two neonatal units in tertiary hospitals and six neonatal units in secondary hospitals in Finland. The units agreed to carry out the training program and its evaluation study. FCC was already used as the guiding principle in the units at some level, and parents could participate in the care of their infant in every unit. In 5 units, however, parents were not allowed to participate in caretaking during the first morning of care because the nurses regarded it as an important hands-on moment to get to know the infants. Parents were not allowed in the room during procedures, either. Four of the units had a family room next to the unit, where parents could stay overnight with their infant when she/he was stable enough and did not need central monitoring. One of the units had only single-family rooms. Aside from the single-family room unit, parents were not allowed to stay overnight in intensive care rooms in any of the units. In two hospitals, neonatal wards also admitted older children. Characteristics of the participating NICUs are presented in Table 1.

**Table 1:** Characteristics of NICUs

Unit	1	2	3	4	5	6	7	8
Patient beds in the unit	5	14	6	10	15	16	20	16
Patients/year	240	400	1400^a^	320	955^a^	1350^a^	400	500
Lower limit of planned deliveries in the hospital (gestational weeks)	32	32	32	30	32	35	22+	22+
Staff								
Nurses	12	24	21	22	28	30	52	50
Neonatologists/pediatricians	2	1	2	2	2	2	4	4

^a^Included also pediatric admissions

### Intervention

The Close Collaboration with Parents Training Program is a systematic education program lasting 12 months with trainer mentors and an additional 6 months independently by the units. It was initially developed and carried out at Turku University Hospital between 2009 and 2012, where it was shown to promote staff skills, positive attitudes toward FCC [14], and decreased depression symptoms among the mothers of very preterm infants [25]. The training consisted of four theoretical phases lasting 4 to 5 months each. The clinical goals of the phases and implementation strategies are presented in Table 2.

**Table 2:** The theoretical phases, the aimed clinical practices, and implementation strategies of the training program

Intervention	Effect on clinical practices	Strategy of implementation
General features		Targeted to the whole multiprofessional NICU healthcare team
0. Pre intervention		Negotiation with leadership • timing • engagement • resources Audit of current practices
1. Phase I: Observation of infant behavior	The staff learn to observe infant behavior to identify each infant’s individual features and preferences; Staff learn to communicate about infants’ individuality	Theoretical education • Lectures • Demonstrations • Learning material (manual) Individual experiential learning • Mentoring: Bed-side observation practices with a mentor • Reflecting on the observation experiences Unit level experiential learning • Reflecting on new understanding or discoveries with colleagues
2. Phase II: Joint observation	Staff learn to actively listen to parents’ perceptions about their infant through joint observations; Collaborative planning of infant care based on joint observations ➔ supporting partnership between staff members and parents	Theoretical education • Lectures • Demonstrations • Learning material (manual) Individual experiential learning • Mentoring: Bed-side joint observation practices with a mentor using ‘See Me Develop’^a^ • Reflection on the observation experiences Unit level experiential learning • Reflecting on new understanding or discoveries with colleagues
3. Phase III: Individual story of the family	Getting to know the individual story of the family and their infant; Developing empathy; Individualized plan about the parental participation in the care of their infant ➔ supporting partnership between staff members and parents	Theoretical education • Lectures • Demonstrations • Learning material (manual) Individual experiential learning • Mentoring: Bed-side semi-structured discussion practices using a modified version of the Clinical Interview for Parents of High-Risk Infants CLIP-I^a^ • Reflecting on the discussion practice with a mentor Unit level experiential learning • Reflecting on new understanding or discoveries with colleagues
4. Phase IV: Family centered transition to home	Collaborative planning of transition to home; Shared decision-making; Including the parents in the healthcare team ➔ supporting the partnership between staff members and parents	Theoretical education • Lectures • Demonstrations • Learning material (manual), ‘Step toward home’^a^ Healthcare team level experiential learning • Medical round observation practices • Reflection of the medical round observation with healthcare team

^a^Tools provided to the staff to practice collaboration with parents during the bedside practices

The strategies for implementing the attitude change and clinical practices included theoretical teaching, practice with mentors, and reflection. Bedside mentoring and reflecting on the experiences were a main part of the learning because individual learning was based on experiential learning theory. The staff were also provided with new tools to learn to recognize the unique qualities of the infants and individuality of parents and families in order to plan an individualized transition to home. These tools helped the staff gain new experiences with families and facilitate their learning. [23]

The training of the mentors and staff occurred as follows: first, two to four registered nurses in the participating units were trained as mentor nurses by the trainer team. The trainer team consisted of master trainers and trainer mentors. The master trainers included an academically trained psychologist, neonatologist, and nurse. The trainer mentors were nurses that had experience with mentoring in their own unit before mentoring another unit. The psychologist of the trainer team supported the trainer mentors in their work. The training started with a one-week theoretical training for all mentor nurses, staff members, and leadership, including lectures, demonstrations, and small group practice. Next, each phase started with the trainer mentors mentoring the units’ mentor nurses, who then implemented this phase in the unit by mentoring the staff of the unit. In the unit, the bedside practices were supported by the training manual. During these bedside practices, the staff learned to collaborate with the parents and support them in their authentic working environment. Staff members participated in group discussions and reflected on their experiences with bedside practices. Detailed descriptions of the content of the phases and implementation strategies are reported in the article by Ahlqvist-Björkroth et al. (2017) [23].

### Participants

Unit managers (doctors and/or head nurses) and nurses were recruited through purposive sampling. They were sent participation requests by e-mail. The head nurse of each NICU gathered the nurses available on that day for the nurses’ interview. The inclusion criterion was that the managers and nurses had already been working in the NICU before the training.

In total, 19 unit managers (five doctors and 14 head nurses) and 32 nurses (registered nurses and licensed practical nurses) participated in this study. Participating nurses included both mentor nurses and bedside nurses. The unit managers’ mean age was 49 years (range: 35–60 years). Six of them were men and 13 women. On average, they had 21 years of working experience (range: 2–34 years). All participating nurses were women. Their mean age was 39 years (range: 26–61 years), with an average of 12 years of working experience (range: 1–30 years).

### Data collection

The data were collected through 16 focus group interviews and two individual semi-structured interviews by one researcher (MT or AA). Two individual interviews were done because two key informants were not able to attend the focus group interviews. Both researchers were familiar with neonatal care and the content of the training program, and they had previous experience conducting interviews. The researchers did not know the participants beforehand and had not participated in the training of the staff.

The focus group interviews and individual interviews were conducted 6 months after the training had ended in the units between August 2014 and June 2017. Each focus group interview was held in the participating NICUs. One individual interview was conducted in the hospital and the other by phone. The managers and nurses were interviewed separately, so two focus group interviews were arranged at each hospital. Separate focus group interviews were chosen to guarantee nurses the possibility to voice their opinion without managers’ presence. The focus group interviews included two to seven people and lasted for an average of 53 min (varying between 13 and 120 min). The focus group interviews and individual interviews were recorded.

Nurses and managers were asked to describe their perceptions of the program and its implementation. The themes (such as parents’ participation, barriers and facilitators of the implementation of training program, and the process of its implementation) guided the focus group interviews as well as the individual interviews. These themes were explored more in-depth with prompting questions, such as “Can you tell me more about what you mean when you say you had to change your professional role?” or “Why do you think that the unit design is challenging regarding new practice?” An additional file shows the focus group interview guide in more detailed. [See Additional file 1.]

### Data analysis

Data were analyzed using deductive thematic content analysis [26]. The Promoting Action on Research Implementation in Health Services integrated framework (i-PARIHS) guided the analysis. The i-PARIHS explains that successful implementation results from the facilitation of innovation with the recipients in their context. Therefore, it provided an applicable theoretical framework to systematically analyze our data. Four core constructs (innovation, recipients, context, and facilitation) were used as main themes in the analysis. The innovation construct contains the characteristics of the evidence being implemented, such as clarity, usability, and feasibility for existing practice. The recipients consist of people who are affected by and influence implementation at both the individual and collective level. This construct highlights the importance of individuals in determining the uptake of new knowledge in practice. The contextual factors encompass the culture of the unit and wider organization. Facilitation refers to the facilitation process in which recipients adopt the innovation within the particular context. The process includes both how and by whom the process is facilitated [27].

The interviews were transcribed verbatim, and all authors carefully familiarized themselves with the data. The first author (MT) then generated codes that captured key statements and thoughts in relation to study aim and collated them into four main themes. The authors (MT, AA, & LL) then discussed the themes and initial subthemes. Fifteen initial subthemes were generated inductively within the main themes. After a careful rereading of the data and discussions among the authors, the initial subthemes were collated to 10 subthemes so that there were no overlaps among them.

## Results

**Fig. 1:**
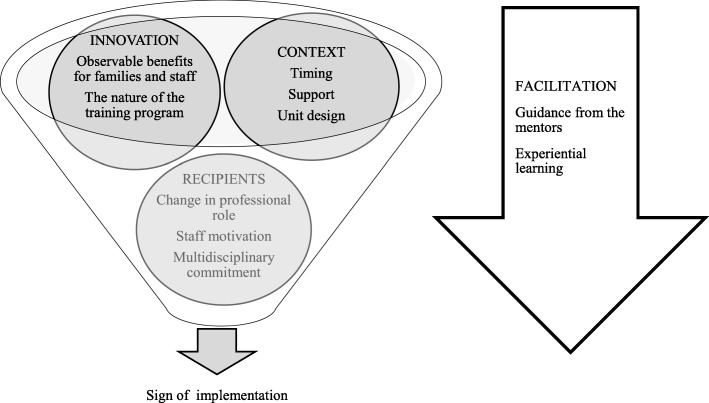
Thematic map, showing findings of the elements affecting the implementation**.** Main themes (innovation, context, and recipients) and subthemes interrelated and influenced each other during the implementation process. Facilitation is one of the main themes but is positioned differently to describe the facilitation process as the element that activates the implementation

The participants of the study identified guidance by the mentors and other experiential learning methods as the main facilitators of the Close Collaboration with Parents Training Program. In addition to the facilitation process, other key elements affecting the implementation were the characteristics of the innovation and its observable benefits; elements concerning the recipients, such as motivation and commitment; and contextual elements, such as support from the leadership, timing, and unit design. Four main themes and 10 subthemes interrelated and influenced each other and either promoted or hindered the implementation (Fig. 1). Findings are organized according to the time order of the implementation progress.

### Context/timing

The unit leadership decided to initiate the training program. Both the nurses and managers commented that the mental preparation for the training and for the changes the training program aimed at was important for the implementation. One of the managers said about timing: *“We had time to psychologically prepare for it [training program] and then we had everything ready for this training” (Managers group a).* In addition, the identified need for a change in care practices to involve parents more in infant daily care promoted the implementation, as two of the managers said: *“We know the practice will be different when we move to the new hospital [with single family rooms], and because of that we had to change our care culture” (Managers group d).* On the other hand, the staff who had no time to prepare for the training felt that it started too quickly, which made the implementation more difficult. *“Our highest leadership just informed that now we start. We should have done it more systematically” (Managers group g).*

### Innovation/the nature of the training program

Both managers and nurses mentioned receiving inadequate initial information about the goals and schedules as a weakness of the training program. It was not until during phase 2 or 3 that they understood how the knowledge of infant observations related to the final goal of the training. *“Our mentor nurses didn’t get a good view of the program as a whole when it started. They only got some crumbs of information” (Managers’ group g).* Some of the staff wished that the training phases had been introduced in shorter time intervals.

### Recipients/change in professional role

The units decided to allow parents’ presence without time limitations after preparation, at the beginning of structured training. Some comments from the managers and nurses indicated that this change caused resistance in some of the nurses. *“I remember that at first some of the nurses said that they will leave. They couldn’t cope with taking care of parents. I think it was because we are used to working routinely and doing the intensive care” (Nurses’ group a).* Participants stated that some nurses were uncomfortable with continuous parental presence at the beginning of the program. They described the feeling that nurses were not needed anymore and of losing their authority. *“First there was a fear that nurses are not needed anymore, but I have not heard those kinds of comments anymore” (Managers’ group h).* They also identified that parental involvement required a change in the nurses’ professional role, which, at first, was seen as difficult. However, the nurses described that they gradually adopted their new role as the parents’ facilitators instead of performing the care by themselves. *“After the training program the nurses’ professional role had changed. I think there is some insecurity about one’s own role regarding the infants’ care” (Nurses’ group a).*

### Facilitation/guidance from the mentors

Two to four mentor nurses implemented the training program in their own unit. Participants emphasized the crucial contribution of the mentor nurses in the progress of the training program and in the changes in care culture. *“Our mentor nurses were motivated. We had a certain resistance here, but nurses anyway gave feedback that their way of working is changing and that parents are more involved” (Managers’ group g). “Well, our mentor nurses deserve the credit for that. They have given 110% of themselves for the training” (Mangers’ group f).* According to the nurses’ and managers’ descriptions, successful mentor nurses were committed, motivated, empathetic, and good communicators. They did not provoke strong or negative emotions, and they had confidence in doing pair practices with colleagues who were more experienced than themselves. *“Our mentor nurses were persons who didn’t stir strong emotions among the work community, and they had a good grip on the training” (Managers’ group h).* On the other hand, the mentor nurses who did not give space to the staff they were training and who did not respect the individual pace and learning ability of their colleagues made mentoring and the implementation of the new care practice difficult. The participants pointed out that the characteristics of a good mentor nurse should be carefully considered when the mentors are chosen. *“Sometimes bad mentoring might spoil a good thing. It’s really important that the right persons are chosen as mentor nurses” (Nurses’ group b).*

### Innovation/observable benefits for families and staff

The staff perceived that the changes in FCC practice were beneficial for infants, parents, and staff alike. That feedback motivated the staff to continue the implementation. Nurses perceived that parents’ increased presence made them more confident in caring for their infant. Closer relationships with parents and increased parent involvement resulted in the transition to home becoming smoother for both the parents and the staff. *“I don’t feel nervous anymore when I discharge the baby, because I know that parents can manage” (Nurses’ group d).* From the doctors’ point of view, the parents made a valuable contribution to their work by knowing their infant well and reporting their observations so they could be used in medical decision-making. *“I get most of the information about the infants’ condition from their parents now and I have noticed that they can give me really valuable information. It’s possible because they are present all the time” (Managers’ group f).*

Participants stated that the training program improved interactions among staff and helped them harmonize care practices. This led to better work satisfaction, as reported by both the managers and the nurses. They also reported that the well-being of the parents and infants was improved. *“Feedback from the parents is like a reward for nurses. They see how good the baby and parents have it together” (Managers’ group d).*

### Recipients/staff motivation

Nurses’ attitudes toward parents became more positive with sustained training. In addition, the staff stated that they had begun to appreciate parents’ presence. *“Our attitude is influencing everything, and that attitude is more permissive now” (Nurses’ group b).* After the training, the nurses perceived parents as more of a resource than a burden in caring for the infants. *“Our attitude has turned to such that we want parents to be here” (Nurses’ group h).* A few comments revealed that the depth and scope of the new attitudes toward parents varied among the units. *“We have really made progress, but it takes time to fully adopt this” (Nurses’ group g). “It is not good if mother stays overnight and then she is too tired to take care of her baby. We have a rule that a mother can stay overnight if she nurses the baby herself” (Nurses’ group g).*

There were differences in adopting the new practice among the nurses. Staff perceived that newly graduated nurses had fewer difficulties in adopting the new care practice because they were not as used to the old one. Some of the nurses who had more work experience felt they occasionally missed the old care practice, especially when the unit was busy and there were a lot of parents. *“Sometimes I wish that parents could visit behind the window. I have experienced that wonderful time, when I got to take care of the infant myself” (Nurses’ group b).* However, some experienced nurses expressed relief at receiving permission to encourage the parents to participate. They had already felt before the training program that parents should not be separated from their infants. *“I don’t have to feel like I’m too kind or soft anymore when everyone has the same agenda” (Nurses’ group d).*

### Recipients/change in professional role

All the interviewed nurses were aware that to ensure implementation, parents’ participation should be voluntary and that every family should decide themselves how much they would attend. *“We have to take into account the situation of the family” (Nurses’ group d)*. Participants revealed that some parents signaled insecurity about their role and about how much time they should spend in the unit. Nurses stated that they wanted to be sensitive to parents’ needs, avoiding burdening them with too much responsibility. *“We have to avoid exhausting parents with too much responsibility” (Nurses’ group h).* It was clear to the nurses that they carried the main responsibility for the infants’ medical care, together with the doctors. Staff recognized that the parents needed support and encouragement to find their role. *“We still represent authority for parents, and if we tell them what to do, they try to do so. We have to encourage them to do what is best for their family” (Managers’ group h).*

### Innovation/the nature of the training program

The training program progressed slowly, which was seen as beneficial by both the managers and nurses. The adaptability and clear structure were also named as strengths of the training program. The program was integrated into the existing practices, and the staff decided themselves how to implement practical changes instead of copying practices from the unit in which the program was developed. Participants stated that the training program provided an analytical way to evaluate the existing practice, thus facilitating the process of learning and moving away from the old practice toward more FCC. *“Family-centered thinking has increased and the whole family is now in the focus, not only the infant” (Managers’ group a).* Various tools used in the training were experienced as helpful to involve the parents in caretaking and to recognize the individual needs of the families. “*I think we have got really good tools and by using them we can learn to know the families” (Nurses’ group h).*

### Facilitation/experiential learning

Experiential learning methods including theoretical teaching, joint observations of infant behavior, and reflections with mentor nurses helped the nurses to recognize the individual needs of infants and to understand the influence of their care on the infants’ behaviors. *“Observing infant behavior was really educational. I learned to watch for different things than before” (Nurses’ group b). “Discussions with the mentor nurse after observations were good” (Nurses’ group d).*

In addition to the discussions with mentor nurses, the interactions among staff also seemed to be important for the implementation. In the units in which the participants stated they had discussed the training program and changes in care practices together, the changes in care culture were found to be more consistent. In the units in which the staff did not agree on changes in care practices, there were more difficulties in implementation. “*We don’t have a uniform approach for care and there should be. You can’t do this job as you like, but we must have the same approach” (Nurses’ group b)*.

### Context/support from the leadership

Nurses and managers expressed that support from the leadership formed the basis for the implementation of the training program. *“In the beginning, we made a common decision that everyone will do their best and all resources that are needed will be given. So we wanted to make sure that we reach the positive outcome and that the change will happen” (Managers’ group f).* Nurses thought that hospital leadership was responsible for allocating enough resources for mentoring. The training process progressed best in the units in which the mentor nurses were given the most work time. *“It has been the most essential factor for succeeding that each of the nurses had time on four days to do infant observations with the mentor in the beginning” (Managers’ group a).* If the mentor nurses had to continue their clinical patient work alongside mentoring, the training was slowed down. *“We didn’t get enough time to do the bedside pair practices even if we told that to a head nurse” (Nurses’ group e)*.

Some of the mentor nurses felt that they carried the responsibility for the success of the training, which was stressful. *“It was really hard. I was so tired at some point. We took care of so many things that I think were not even our responsibility” (Nurses’ group e).* In addition to support from the leadership, the mentor nurses pointed out that the competency of the trainer mentors and master trainers and their supportive approach were important to them. The trainers could provide peer support and advice on how to best facilitate the implementation. *“The trainer mentors supported us even when we didn’t get support from managers” (Nurses’ group e).*

### Context/unit design

Participants stated that unit design complicated the implementation because patients’ rooms were not optimal regarding parents’ presence. *“Parents attend much more but our rooms are not suitable for that. It causes conflicts because we don’t have rooms” (Nurses’ group b).*

### Recipients/multidisciplinary commitment

Nurses reported that the implementation was difficult if the doctors were not committed to the training program. Nurses stated that in that case, they were powerless to help the parents’ voices to be heard. *“Our physicians were not engaged in the program and it made our work difficult” (Nurses’ group g).* The nurses believed that the hierarchy between doctors and nurses could impede mentor nurses from mentoring the doctors, and they suggested that doctors might have been more involved if they had other doctors as their mentors. Participants perceived that the multidisciplinary commitment of staff was important for the success of the implementation. *“The fact is that our staff is behind this. They had to stretch and make this possible” (Managers’ group f).* Commitment was expressed as shared views on the desired change and the determination to succeed in the implementation. *“I think the only way to implement this is that everyone works together for a common goal and knows what we are talking about” (Managers’ group b).*

The elements affecting the implementation of the Close Collaboration with Parents Training Program were similar, even if the units differed from each other regarding architecture and size. The shift in care culture toward more FCC happened in each unit, but the depth and scope of the new attitudes and practice varied among the units.

## Discussion

In this study, we identified key factors affecting implementation of an intervention to change staff attitudes and values and, thereby, to develop FCC in a NICU. We identified elements within all categories presented in the i-PARISH framework based on the data from the 8 units that carried out the Close Collaboration with Parents Training Program. In the following discussion, we examine our findings from the perspectives of the four categories: recipients, context, innovation, and facilitation [27].

### Recipients

According the i-PARIHS framework, the attitudes, motivation, and thoughts of the recipients influence the way new knowledge will be adopted at both the individual and collective team levels [27, 28]. Recipients in this study were the head nurses, bedside nurses, mentor nurses and doctors of the participating units. Implementation of new care practices reshaped the nurses’ and doctors’ role: They gave more space to the parents and adopted a more receptive attitude toward them, although individual variation still remained. Changing routines is challenging and requires a shift in thinking [12], which was shown to be difficult for some individuals. Similar to our findings, other studies have also reported challenges in changing nurses’ professional role and motivation when implementing elements of the FCC [13, 14, 17, 29]. Therefore, it is crucial that the staff is prepared for this change in their professional role [10, 30, 31].

Our study emphasized that a multidisciplinary approach was essential for the progress of the implementation. The implementation of the training program progressed best in the NICUs in which doctors were also committed to the program. Without their commitment, the team did not consistently and coherently work together with parents. According to the literature, complex interprofessional relationships and the hierarchy between doctors and nurses could hinder implementation of new knowledge [28, 32]. The significance of a multidisciplinary approach has also been shown in other implementation studies in relation to kangaroo mother care (KMC) and baby-friendly care in NICUs [13, 29].

### Context

This study identified many facilitating or hindering contextual elements including the recognition of the need for a change, preparedness, leadership support, and unit design. These elements have also been identified in other implementation articles [12, 21, 27, 32] including those aiming to increase baby-friendly care and KMC in NICUs [13, 29] .

The leadership was responsible for choosing the optimal timing for the training, allocation of resources, and supporting the mentors in their unit. Leaders should create a good foundation and preparedness for a change [12, 28], including adequate staff resources. It has been stated that half of organizational changes fail because of leaders’ failure to establish sufficient organizational readiness for change [12].

### Innovation

It is challenging to develop an intervention that works in a variety of contexts [21]. In our study, the participants perceived that the Close Collaboration with Parents Training Program was adaptable to various contexts because the training focused on changing attitudes, and the care practice changes were decided by the staff themselves. In this way, the practice changes were tailored according to the context. Bedside practice promoted the integration of the new knowledge into everyday work. The training program succeeded in being respectful and indicated appreciation for the staff in each unit by empowering them to become the change agents. The i-PARIHS model argues that if new knowledge can be tailored to existing practice, it promotes the implementation [27]. The staff received immediate positive feedback from the infants and parents as they learned to understand the behavioral cues of the infants and to listen to the parents. The observable results have shown to be of major significance for implementation success [27, 29]. It is important to ensure the feedback loop for the staff.

### Facilitation

The mentor nurses were essential facilitators of the implementation process, as reported by both the managers and nurses. The characteristics of the mentor nurses influenced the way the training program progressed in the units. Our study emphasizes the importance of the recruitment process of the mentor nurses. Recruiting the wrong mentor nurse could even hinder the implementation according to the experiences of both nurses and managers in our study. Voluntary-based recruitment is not an optimal approach for choosing a mentor nurse. Instead, characteristics such as flexibility, responsiveness, motivation to make the changes, and enthusiastic encouragement of mentees are important [27, 33, 34]. A mentor nurse should provide space so that the staff members can make their own observations and draw their own conclusions. The role of a mentor is to help and enable rather than to tell or persuade [35].

## Strengths and limitations

These results are transferable to other neonatal units in similar settings in high-income countries. The implementation of the training program in other settings requires further research. Focus group interviews with a large number of participants provided rich data because colleagues completed each other’s thoughts and reflected on the implementation together. Even if data saturation did not guide the data collection in our study, saturation was achieved after the focus group interviews in 6 units, supporting the trustworthiness of our findings. The face validity of our findings is supported by participants from four NICUs who reviewed them. A limitation of the study was that it was not possible to include doctors from all NICUs. The findings represent the subjective experiences of nurses and managers, while parents’ experiences were not examined in this study. In the future, we plan to explore parents’ perspective on the quality of FCC before and after the Close Collaboration with Parents training program. In the future, the findings of this study can help us to scale up the implementation of the training program and evaluate its effectiveness. Effects among units might differ, although the intervention and the implementation strategies do not vary. The intervention interacts with its context, which is always highly complex [24].

## Conclusion

Elements that appear to have been positive to implementation were flexible and motivated mentor nurses who supported the change, team commitment of the whole unit and support from the leadership. Barriers to implementation included inadequate preparation for the change and non-supportive unit design.

## Additional file

Additional file 1: Focus group interview guide. Themes and questions used to guide focus group interviews (DOCX 15 kb)

## Data Availability

The datasets used and analyzed during the current study are available from the corresponding author on reasonable request.

## Abbreviations

FCC: Family-centered care
KMC: Kangaroo mother care
NICU: Neonatal intensive care unit
