# Supplementary material for: Key factors supporting implementation of a training program for neonatal family- centered care – a qualitative study
Source: BMC Health Serv Res. 2019 Jun 19;19:394. doi: 10.1186/s12913-019-4256-1 (PMC6585011; doi:10.1186/s12913-019-4256-1)
Supplement: Supplementary file 1 — Focus group interview guide. Themes and questions used to guide focus group interviews (DOCX 15 kb) [file 12913_2019_4256_MOESM1_ESM.docx]

Additional file 1. Focus group interview guide

Themes and questions used to guide focus group interviews

1. What do you think the Close Collaboration with Parents training program changed in your unit’s practices or policies?
2. Are parents allowed to do something more with their infant than before the training program?
3. How did the training program progress or work?
4. What were the facilitators or barriers, that had an effect on the implementation of the training program?
5. If the training started now, is there anything you would do differently?
